# Spectroscopy of Laser-Induced Dielectric Breakdown Plasma in Mixtures of Air with Inert Gases Ar, He, Kr, and Xe

**DOI:** 10.3390/s23020932

**Published:** 2023-01-13

**Authors:** Andrew Martusevich, Roman Kornev, Artur Ermakov, Igor Gornushkin, Vladimir Nazarov, Lyubov Shabarova, Vladimir Shkrunin

**Affiliations:** 1 Laboratory of Translational Free Radical Biomedicine, Sechenov University, 119991 Moscow, Russia; 2 Laboratory of Medical Biophysics, Privolzhsky Research Medical University, 603005 Nizhny Novgorod, Russia; 3 Institute of Chemistry of High-Purity Substances, 603951 Nizhny Novgorod, Russia; 4 BAM Federal Institute for Materials Research and Testing, 12489 Berlin, Germany

**Keywords:** laser-induced dielectric breakdown (LIDB), nitrogen monoxide, nitrogen dioxide, ozone, emission spectroscopy, inert gases, thermodynamic analysis

## Abstract

The generation of ozone and nitrogen oxides by laser-induced dielectric breakdown (LIDB) in mixtures of air with noble gases Ar, He, Kr, and Xe is investigated using OES and IR spectroscopy, mass spectrometry, and absorption spectrophotometry. It is shown that the formation of NO and NO_2_ noticeably depends on the type of inert gas; the more complex electronic configuration and the lower ionization potential of the inert gas led to increased production of NO and NO_2_. The formation of ozone occurs mainly due to the photolytic reaction outside the gas discharge zone. Equilibrium thermodynamic analysis showed that the formation of NO in mixtures of air with inert gases does not depend on the choice of an inert gas, while the equilibrium concentration of the NO+ ion decreases with increasing complexity of the electronic configuration of an inert gas.

## 1. Introduction

The study of the biological and therapeutic effect of such gas mediators as ozone (O3) and nitrogen monoxide (NO) is an urgent task; therefore, the issues of personalization of ozone therapy and control of its effectiveness, studies of the biological activity, and sanogenetic effects of NO are relevant.

Despite the fact that ozone, an allotropic form of oxygen, has been widely used in medicine for more than a hundred years, new possibilities for its use are opening up. The biological effect of ozone includes an immunomodulatory property, an increase in cellular energy, and an increase in antioxidant protection [1]. The possibilities of using ozone in dental practice [2], autohemotherapy in the form of an ozonized sorbent [3], and rectal infusion [4] are widely presented.

Another actively developing direction is the use of nitric oxide in medical practice, for example, for the treatment of pulmonary diseases. In addition, several effects of singlet oxygen therapy [5,6,7,8] are associated with the presence of NO in the gas flow of the singlet oxygen generator. Among the functional effects, one can note the activation of blood flow through small-diameter vessels (capillary bed) [7], the activation of antioxidant expansion without stimulating free radical oxidation [9,10], the enhancement of energy reduction and detoxification of cells, the enzymes in them [5,6], etc.

As shown in review [11], it is convenient and economically expedient to synthesize NO or O_3_ in situ from air using a plasma process. Barrier discharges are often used to generate ozone [12,13,14,15,16,17]. Garamoon et al. [17] studied the formation of ozone in an oxygen–air barrier discharge and it was found that the concentration of O_3_ increases by more than three times when the pressure decreases by two times. It was also found that increasing the length of the gas discharge tube, the interelectrode distance, and the applied potential also increases the ozone yield.

Moreover, ICP plasma and microwave discharges can be used to produce O_3_. Fuller et al. [18] determined the conditions for the formation of metastable active O and O_2_ particles responsible for ozone generation, and it was shown that their concentrations depend on the pressure and discharge power, while the amount of dissociated oxygen does not exceed 2%. Cvelbar et al. [19] studied the emission spectra of an oxygen-containing ICP at pressures of 10–300 Pa. The emission spectra contains lines of atomic oxygen at 438.8, 615.6, 645.4, 777.2, and 844.6 nm, and molecular bands of metastable O_2_ ($b^{1}\Sigma_{u}^{+}$) at 760.5 nm and O_2_^+^ ($b^{4}\Sigma_{g}^{-}$—$a^{4}\Pi_{u}$); these particles presumably contributed to the formation of ozone. Pulsed and stationary microwave discharges were used in [20] to study the formation of nitrogen oxides; strong NO production and low NO_2_ production was demonstrated.

An additional important requirement for medical gases is their purity. At the same time, the gas treated with discharges with metal electrodes can be contaminated with metal particles resulting from spark erosion of the electrodes or cathode sputtering. These impurities are characteristic of barrier and spark discharges, while in ICP impurities can come from the surface of a quartz tube upon contact with the ICP plasma. Therefore, it is important to look for the type of discharge in which these processes are excluded. One of the promising discharges capable of generating ozone and NO without contamination is the optical gas breakdown by a high-power laser, the so-called laser-induced dielectric breakdown (LIDB). 

Stricker [21] studied the characteristics of laser breakdown in pure N_2_ and O_2_ in the pressure range from 1 to 50 atm using a Nd:YAG laser with a wavelength of 1.064 μm and a pulse duration of 10 ns. The emission spectra revealed the presence of O^++^ and O^+^ with lifetimes of 15–20 ns and 2 µs, respectively, while no O or O_2_ emission was observed. Svatopluk et al. [22] studied the breakdown of CO-N_2_-H_2_O gas mixtures using a near-IR laser with a power of 85 J and a pulse duration of 450 ps. The various stages of the discharge were studied using time-resolved OES and gas composition using FTIR and GC. The presence of N_2_O, CO_2_, ethane, acetylene, ethylene, and acetone was detected in the mixture after its laser irradiation. Gornushkin et al. [23] studied the formation of ozone and nitrogen oxides during multiple laser breakdown of oxygen–nitrogen mixtures at atmospheric pressure. About 2000 laser pulses from a Nd:YAG with irradiance of 10^10^ W cm^−2^ were fed into the sealed reaction chamber. The chamber with a long capillary was designed to measure the absorption of O_3_, NO, and NO_2_ depending on the number of laser pulses. The source of light for measuring the absorption was continuous radiation emitted by the plasma during the first 0.2 µs of its evolution. A kinetic model has been developed that takes into account the chemical reactions between atmospheric components and laser breakdown products. In the model, the laser plasma was considered as a source of nitric oxide and atomic oxygen, the formation rate of which was calculated based on the measured absorption of NO, NO_2_, and O_3_. The calculated NO, NO_2_, and O_3_ concentration profiles were in good agreement with the measured profiles on the time scale of 0–200 s; the calculated yield of 2 × 10^12^ ozone molecules was consistent with the model prediction. This study was important for a general understanding of the chemistry of laser plasmas and for elucidating the nature of spectral interferences and matrix effects important in a spectrochemical analysis. 

The aim of this work is to study the spectral features of LIDB plasma in a mixture of air with Ar, He, Kr, and Xe and their influence on the synthesis of NO, NO_2_, and O_3_. In the future, we plan to use this technology for medical purposes. Air is offered as the most accessible precursor that does not require additional costs from a potential customer (for example, additional gas cylinders), and also reduces the risks associated with the use of gas equipment (working with high-pressure oxygen is dangerous). Inert gases that do not form stable compounds with air components are chosen as electron sources.

## 2. Materials and Methods

**Figure 1:**
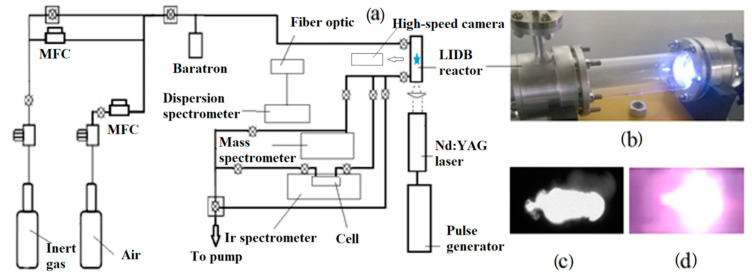
(**a**) Schematic of experimental setup; (**b**) LIDB reactor; (**c**) image of the discharge recorded by camera Nanogate 24/3; (**d**) photograph of the discharge.

The experimental setup is shown in Figure 1a. This allows a comparative study of the plasma generated by LIDB in various gas mixtures using OES, IR, and mass spectrometry. The plasma is created inside a quartz cylinder 40 mm in diameter, placed in a gas-tight stainless-steel chamber with quartz windows for input and output of laser radiation and registration of radiation spectra (Figure 1b). The window for recording IR spectra is made of zinc selenide (ZnSe). To create plasma, an Nd:YAG laser is used with the parameters shown in Table 1. Before filling the chamber with gases, it is pumped out by fore vacuum and turbomolecular pumps (not shown in Figure 1a). The dosing of inert (Ar, He, Ne, and Kr) and working (air, CO_2_) gases is controlled by precision gas flow regulators (RRGs). To study the synthesis of active particles, the UV-Vis, IR, and mass spectrometers were connected to the setup. A high-sensitivity Nanogate 24/3 camera was used to record the plasma light. The camera was located at 30 cm from the reactor wall, perpendicular to its cylindrical axis.

### 2.1. Emission Spectroscopy

Optical emission spectroscopy (OES) was used to detect reaction products in plasma. Emission spectra were recorded by an AvaSpec-ULS2048CLEVO-RM-USB3 multichannel 2048-pixel fiber optic spectrometer with ultra-low light scattering. The spectra were recorded in the range 217.5–710 nm with a resolution of 0.17 nm. The spectrometer was activated by a TTL pulse coming from the laser. Time-resolved spectra were obtained by shifting the delay time of the start of recording subsequent measurements by 1 µs, up to 270 µs, when only noise could be recorded. The signal accumulation time varied from 30 to 60 μs.

### 2.2. IR Spectroscopy 

The IR spectra of gas mixtures before and after laser irradiation were recorded in the range 450–7000 cm^–1^ on a BrukerVertex 80v spectrometer with a DTGS detector with a resolution of 1 cm^–1^. For this, we used an IR gas cell with an optical path length of 10 cm (see Figure 1a) and pressure inside the cell from several tens to several hundreds of Torr. The windows for recording IR spectra in the IR cell are made of zinc selenide (ZnSe).

### 2.3. Mass Spectrometry of Gases 

Mass spectra of gaseous mixtures before and after laser irradiation were recorded on an ExtorrXT300(M) SeriesRGA quadrupole mass spectrometer with a resolution of 1 amu. The residual vacuum in the mass spectrometer chamber was 5 × 10^−8^ Torr. The working pressure was varied from 1 × 10^−6^ to 1 × 10^−5^ Torr to observe the mass spectra of gas mixtures with different concentrations of components. The pressure was controlled by pressure sensors of the mass spectrometer and normalized to the band of the Ar^+^ carrier gas, which did not take part in the reaction.

### 2.4. Spectrophotometry of Gases

The absorption spectra were recorded with an SF-2000 spectrophotometer before and after laser irradiation at time intervals of 0 and 10 min in the range of 200–1100 nm with an exposure time of 0.2 s with a resolution of 1 nm. The resulting signal was displayed after fifteen accumulation cycles. The spectrophotometric analysis was carried out by introducing 1 atmosphere of a mixture of air and an inert gas in a ratio of 1:1 into a cylindrical cell-reactor made of fluoroplast with plane-parallel quartz glasses. Before irradiation with the Nd:YAG laser, the spectrum of each of the mixtures was recorded as a blank experiment. 

Ozone concentration was calculated using the Hartley band (200–350 nm) with a maximum at λ = 254 nm. The absorption cross section of the remaining components present in our reaction mixture in this wavelength range was more than two orders of magnitude lower. The NO_2_ concentration was calculated in the range of 350–430 nm at the maximum λ = 400 nm. This region of the spectrum is transparent for ozone. The concentrations were calculated based on the Bouguer–Lambert–Beer law. The molar extinction coefficients of 3000 M^−1^·cm^−1^ for O_3_ at 254 nm and 157 M^−1^·cm^−1^ for NO_2_ at 400 nm were taken from the database [24].

### 2.5. Thermodynamic Analysis of the System Air + Ar (Kr, Xe)

To estimate the possible yield of ozone and nitrogen oxides in the LIDB plasma, a thermodynamic analysis of gas mixtures was carried out. The calculations were performed out using open-source software [25], which implies local thermodynamic equilibrium (LTE) and is based on the Gibbs free energy minimization algorithm. This model assumes the equation of state for an ideal gas and allows for a small amount of condensed phase due to its negligible volume compared to gaseous particles. The question of whether LTE occurs in LIDB plasma is rather controversial. It is generally accepted that LTE is possible at intermediate and late stages of plasma plume development, somewhere between 1–20 µs, when the characteristic times of the hydrodynamic flow become larger than the characteristic time for chemical reactions. A more complex set of conditions under which a plasma can be considered close to LTE is given in [26,27].

## 3. Results and Discussion

### 3.1. Emission Spectra of Ar, He, Kr, Xe in LIDB Plasma

**Figure 2:**
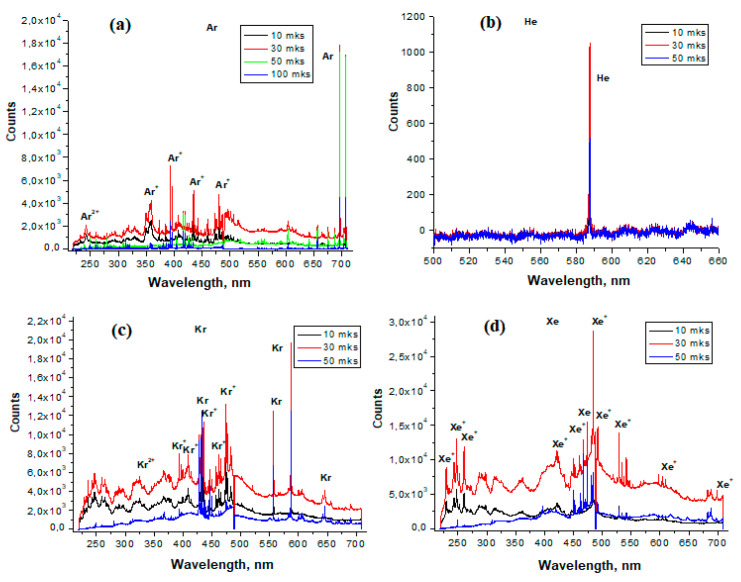
Emission spectra of LIDB plasma in: (**a**) Ar; (**b**) He at 2 atm; (**c**) Kr; (**d**) Xe.

The main factors affecting the plasma lifetime are the electron configuration, ionization potential, and the presence of metastable states of plasma particles. Figure 2 shows the emission spectra of pure He, Ar, Kr, and Xe. The electronic configuration of He is simple compared to other inert gases; it has many electronic levels near a very high (24.6 eV) ionization potential (Table 2) [28]. It was difficult to create a laser breakdown of helium at atmospheric pressure because of its low density; this was possible only at a pressure of 2 atm. The existence of helium plasma is largely due to the presence of a metas Table 2 ^3^S_1_ level with a lifetime of 6 × 10^5^ s (Table 3). In the emission spectrum of He, shown in Figure 2b, only one line is visible at 587 nm, corresponding to the transition between levels with energies of ~23 eV and ~21 eV [29].

The emission spectra of Ar, Kr, and Xe have many lines in the UV, visible, and IR regions (Figure 2a,c,d). This is due to their electronic configuration with many electronic levels (Table 2) distributed between ground state and ionization potential. The intensity of the lines in the emission spectrum of the argon plasma shows that its lifetime is more than 100 μs. Most likely, this is due to the presence of long-lived metastable Ar states with energies of 11.55 and 11.72 eV and a lifetime of more than 1.3 s (Table 2). For He, Kr, and Xe plasmas, the signal drops to the noise level at times longer than 50 μs.

**Figure 3:**
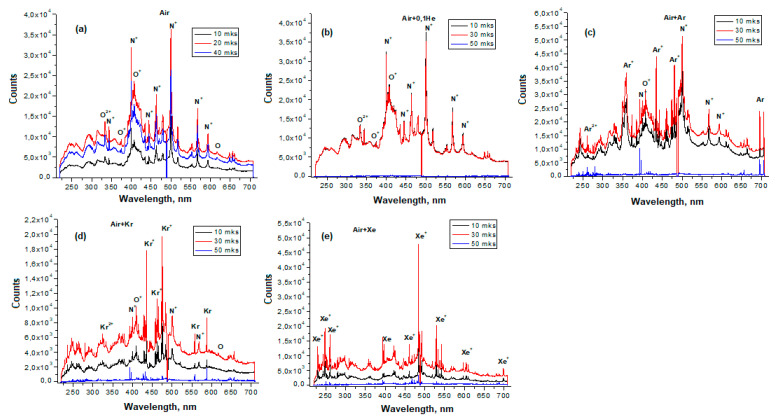
Emission spectra of air and its mixtures with inert gases in LIDB plasma: (**a**) pure Air; (**b**) Air + He; (**c**) Air + Ar; (**d**) Air + Kr; (**e**) Air + Xe.

Figure 3 shows the emission spectra of pure air, as well as its mixtures with Ar, He, Kr, and Xe. In the emission spectrum of air (Figure 3a), noticeable lines of a singly-charged nitrogen ion appear; oxygen is represented by both an atomic line and lines of ions O^+^ and O_2_^+^. Most of the spectral features disappear after 40 µs, only weak transitions of atomic oxygen at 615.8 nm and the O^+^ ion (640–660 nm) are preserved. After 50 µs, only noise is observed.

When analyzing the spectrum of the Air + He mixture (Figure 3b), the overall picture of the spectrum does not noticeably change, the spectral features of pure air also remain. It should be noted that the formation of a gas discharge is observed at a ratio of Air/He ≥ 9. At a higher concentration of He in air, the discharge is not initiated. The line of helium itself is not observed in the emission spectrum. In this regard, it can be assumed that the observed plasma is formed by the air components O_2_ and N_2_, whose ionization potentials (Table 2) are 12.2 and 15.6 eV, respectively, and the configuration of electronic levels is complex [30]. Thus, He does not affect the composition of the reactive components of the Air + He mixture.

**Figure 4:**
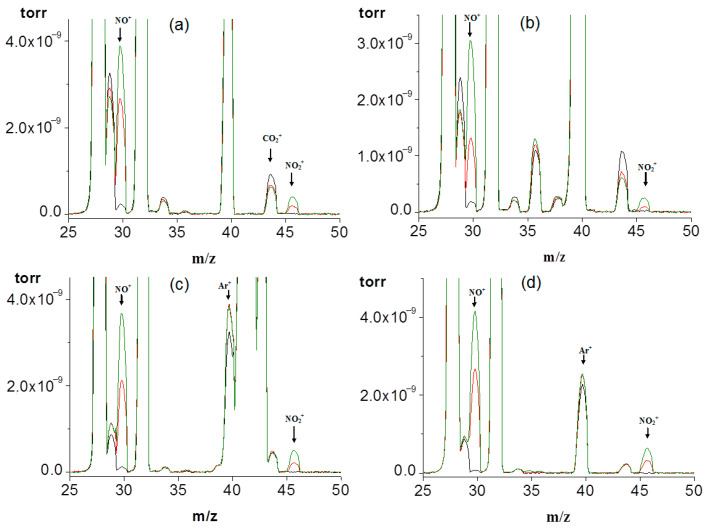
Mass spectra of mixtures of inert gases and air before and after exposure to LIDB plasma: (**a**) Air + He; (**b**) Air + Ar; (**c**) Air + Kr; (**d**) Air + Xe. Black line—before plasma exposure, red line—after 1 h of plasma treatment, green line—after 2 h of plasma treatment.

Argon makes it possible to extend the glow time of the discharge of the Air/Ar = 1 mixture up to 80 µs (Figure 4c). The spectrum is rich in lines of atomic and ionized argon. As well as in the case of pure air, N^+^ and O^+^ transitions were recorded. The intensity of the nitrogen line N^+^ at 500.5 nm is noticeably higher than in the previous cases. The intensity of the N^+^ line at 399.5 nm is lower, and the N^+^ line at 463.0 nm is not observed. The lines of ionized oxygen are intensified.

For the Air/Kr = 1 mixture (Figure 3d), the plasma lifetime is 50 µs, and the spectrum pattern noticeably changes. The most intense are the lines of singly-charged krypton at 473.9 nm and 435.5 nm. The lines of air components noticeably decrease, the N^+^ line at 594.1 nm is absent, and the bands of ionized oxygen O_2_^+^ disappear.

The emission spectrum of the Air/Xe = 1 mixture (Figure 3e) contains mainly xenon lines. Of the air components, only a weak N^+^ line at 399.5 nm is observed. The most intense Xe^+^ line is observed at 484.3 nm, and the plasma lifetime is 60 µs. 

Figure 3b–e shows that only lines of air components are observed in the Air + He mixture. In Air + Ar and Air + Kr mixtures, both lines of air components and lines of inert gases appear. In an Air + Xe mixture, the Xe lines are visible, and the lines of the air components are represented by a weak N^+^ line.

It can be assumed that the ionization of helium is very difficult due to the high ionization potential, and the energy is directed to the ionization of air. Xe ionizes more easily than other inert gases; energy is spent on its ionization and excitation of its metastable states. Table 3 presents the comparative intensities of the lines of excited air particles in accordance with the above considerations.

Since the plasma produced by LIDB is close to equilibrium, gas ionization occurs not only due to electron impact, but also due to the impact of heavy particles. Therefore, the following reactions can be written:

(1) $$N_{2}\overset{t}{\to }\;2N$$

(2) $$O_{2}\;\overset{t}{\to }\;2O$$

(3) $$N_{2\;}\overset{t}{\to }\;2N^{+}+2e$$

(4) $$O_{2}\;\overset{t}{\to }\;2O^{+}+2e$$

(5) $$O_{2}\;\overset{t}{\to }\;2O^{2+}+4e$$

(6) $$N\;\overset{t}{\to }{\;N}^{+}+e\;$$

(7) $$O\;\overset{t}{\to }{\;O}^{+}+e$$

(8) $$O\;\overset{t}{\to }{\;O}^{2+}+2e$$

e + N_2_ → 2N^+^ + 2e (9)

e + O_2_ → 2O^+^ + 2e (10)

e + O_2_ → 2O^2+^ + 3e (11)

e + N → N^+^ + 2e (12)

e + O → O^+^ + 2e (13)

e + O → O^2+^ + 3e (14)

Figure 4 shows the mass spectra of mixtures of air with inert gases before and after exposure to LIDB plasma. In all the presented mass spectra, the bands of N_2_ and O_2_ included in the air, as well as NO^+^ and NO_2_^+^ ions corresponding to NO and NO_2_ molecules, are identified. The ratio of the relative intensity of the NO^+^ and NO_2_^+^ fragments in the mass spectrum of the nitrogen dioxide molecule, according to the reference tables provided by the suppliers of equipment for mass spectrometry, is 100:37. In the mass spectra obtained in this work, this ratio fluctuates within 100:(7÷17), from which it follows that both NO_2_ and NO molecules are formed during the reaction according to the following schemes:

N + O → NO(15)

N + 2O → NO_2_ (16)

NO + О → NO_2_ (17)

е + N^+^ + O → NO (18)

е + N + O^+^ → NO (19)

2е + N^+^ + O^+^ → NO (20)

е + NО + O^+^ → NO_2_ (21)

The peak of the NO^+^ ion in the recorded mass spectra consists of the contribution of the NO^+^ ion of the NO molecule present in the air before the plasma-chemical reaction, the contribution of the NO^+^ ion of the NO molecule formed during the plasma-chemical reaction, and the contribution of the NO^+^ fragment from the NO_2_ molecule formed during the plasma-chemical reaction. The relative intensity of the NO^+^ ion related to the NO molecule was calculated as the difference between the intensities before and after irradiation and the contribution of the NO^+^ fragment from the NO_2_ molecule to the intensity of this peak.

**Figure 5:**
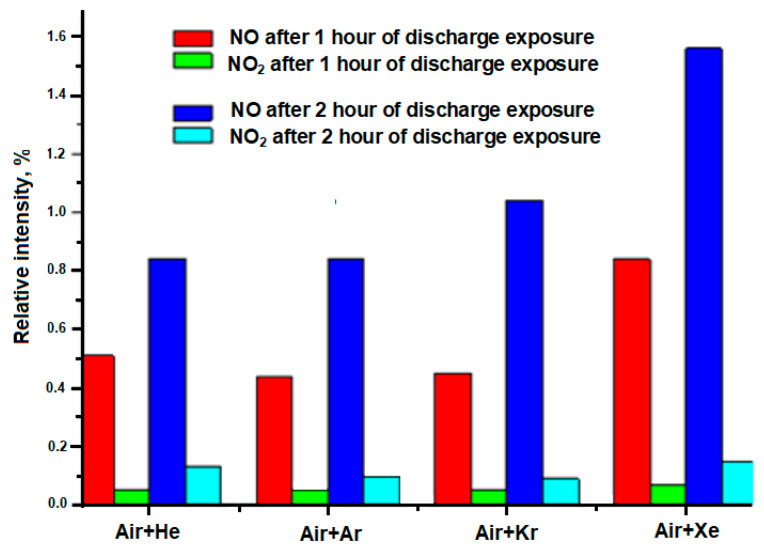
The dependence of the relative intensity of the NO and NO_2_ bands after 1 and 2 h of irradiation of the initial mixture.

The histogram in Figure 5 shows the dependence of the relative intensity of the NO and NO_2_ bands after treatment for 1 and 2 h on the composition of the initial mixture. It can be seen that, depending on the complexity of the electronic configuration of an inert gas, as well as a decrease in its ionization potential, there is a tendency for an increase in the NO compound in the reaction products. No pronounced dependence is observed for the NO_2_ compound.

Of course, the factors that determine the influence of an inert gas on the chemical reactions occurring in the LIDB plasma are not limited to the complexity of the electronic configuration alone. The density of the gas, which increases with increasing atomic mass, can also have an effect. The processes of Pfennig ionization play a role, which are different for different inert gases, as well as the size of the atom; for xenon it is maximum, which means that the cross section of collisions with atoms and electrons is larger than for other inert gases. However, we can assume that these factors are also determined by the structure of the atom, and their influence can be considered separately in the appropriate statement of the problem.

**Figure 6:**
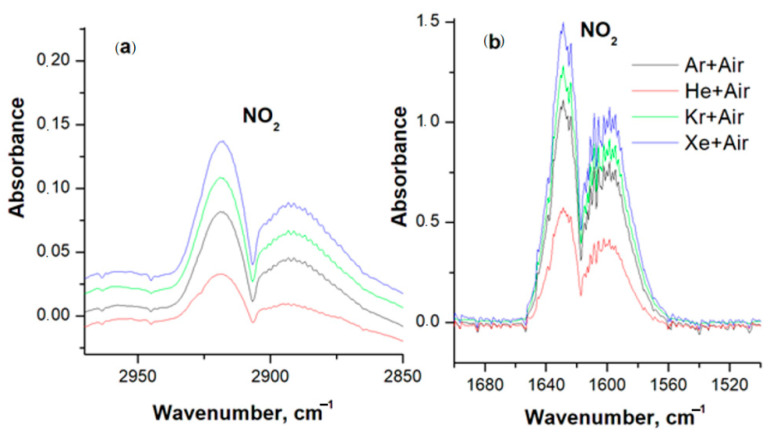
IR spectra of air mixtures with Ar, He, Kr, and Xe. (**a**) nitrogen dioxide absorption bands in the ranges of 2860–2978 cm^−1^; (**b**) nitrogen dioxide absorption bands in the ranges of 1564–1650 cm^−1^.

Figure 6 shows IR spectra of mixtures of air with Ar, He, Kr, and Xe, which confirm the formation of NO_2_. For all mixtures, the spectra after gas discharge treatment contain nitrogen dioxide absorption bands in the ranges of 2860–2978 (Figure 6a) and 1564–1650 cm^−1^ (Figure 6b) [25]. The intensity of the NO_2_ bands in the spectra increases depending on the complexity of the electronic configuration of the inert gas, which agrees with the mass spectrometry data. The lowest intensity of the NO_2_ band is recorded in the Air + 0.1He mixture, the highest in Air + Xe.

It can be seen that, despite the fact that the lines of excited air components are not resolved in the emission spectrum of the Air + Xe mixture due to their low intensity, as well as the insufficient resolution of the spectrometer, the reaction with formation of NO and NO_2_ proceed. We can assume a change in the mechanism of formation of these compounds depending on the gas mixture. The atomic (15)–(17) and ionic (18)–(21) mechanisms of NO and NO_2_ formation in pure air, air + Ar, and air + Kr change to the molecular mechanism (22), (23) in air + Xe:

N_2_ + 2О_2_ → 2NO_2_ (22)

N_2_ + О_2_ → 2NO (23)

**Figure 7:**
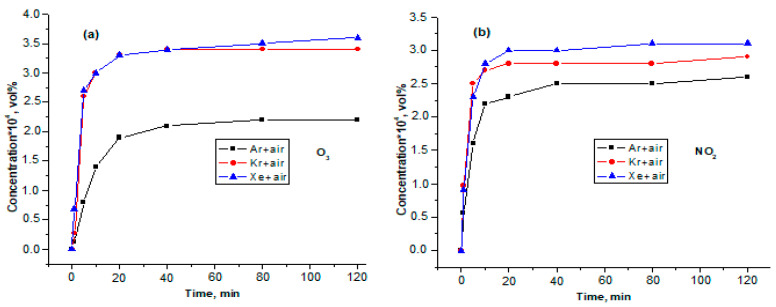
Dependence of O_3_ (**a**) and NO_2_ (**b**) concentrations on plasma treatment time of air mixtures with Ar, He, Kr and Xe.

Figure 7 shows the concentrations of O_3_ and NO_2_ as a function of time during LIDB plasma treatment of mixtures of air with argon, xenon, and krypton. In the case of the Air + Xe mixture, there is a jump in the concentrations of O_3_ and NO_2_ up to the values (3.0 ± 0.2) × 10^−4^ and (2.8 ± 0.2 × 10^−2^ vol.%, respectively, in the first ten minutes of irradiation. Further growth of concentrations slows down and by 2 h of irradiation their values are (3.6 ± 0.1) × 10^−4^ vol.% for ozone and (3.1 ± 0.2) × 10^−2^ vol.% for NO_2_.

A similar situation is observed for the Air + Kr mixture, a sharp increase in the concentrations of O_3_ and NO_2_ within 10 min to the values (3.0 ± 0.2) × 10^−4^ vol.% (O_3_) and (2.8 ± 0.2) × 10^−2^ vol.% (NO_2_). However, after two hours of irradiation, the Air + Kr mixture produces lower amounts of ozone and nitrogen dioxide, (3.4 ± 0.2) × 10^−4^ and (2.9 ± 0.2) × 10^−2^ vol.%, respectively. A tendency to an even lower yield of O_3_ and NO_2_ manifests itself when passing from krypton to argon, where the ozone concentration in the Air + Ar mixture after two hours of irradiation is (2.2 ± 0.3) × 10^−4^ vol.%, and the nitric oxide concentration (2.6 ± 0.2) × 10^−2^ vol.%.

Possible mechanisms of NO_2_ formation were discussed above. The formation of ozone, according to [23], cannot occur in the gas discharge zone, since, due to instability, the O_3_ molecule immediately reacts with N. The most likely mechanism for its formation is the photolysis reaction occurring in the volume of the LIDB reactor according to the following scheme:

hν + O_2_ + O → O_3_ (24)

### 3.2. Thermodynamics

**Figure 8:**
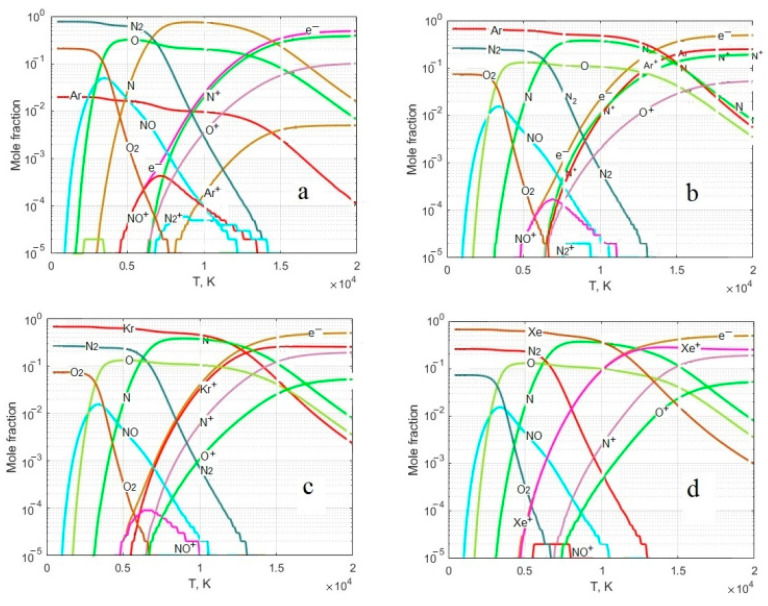
The equilibrium composition of: (**a**) air (N:O:Ar = 78:21:1) and its mixtures; (**b**) N:O:Ar = 39:11:50; (**c**) N:O:Kr = 39:11:50; (**d**) N:O:Xe = 39:11:50. P = 1 bar.

Considering that the LIDB plasma at atmospheric pressure is close to equilibrium, thermodynamic analysis can be used to consider plasma-chemical reactions. Figure 8 shows the thermodynamically equilibrium composition of air, as well as its mixtures with Ar, He, Kr and Xe. Analyzing the dependences obtained, it can be seen that in pure air, the formation of O^+^ and N^+^ ions begins at temperatures above 6000 K. Their presence is seen in the emission spectrum (Figure 3a). For Air + Ar and Air + Kr mixtures, the formation of O^+^ and N^+^ ions also begins from a temperature above 6000 K, but at the same time, the Kr^+^ ion in the Air + Kr mixture is formed at T = 5400 K, while the Ar^+^ ion, in the Air + Ar mixture, at T = 6000 K. For the Air + Xe mixture, the formation temperature of the Xe^+^ ion decreases to 4500 K, while the formation of O^+^ and N^+^ ions also starts from a temperature above 6000 K.

However, while in the emission spectra of Air + Ar and Air + Kr mixtures, one can see O^+^ and N^+^ ions together with Ar^+^ and Kr^+^ ions, in the emission spectrum of Air + Xe mixtures, no O^+^ and N^+^ ions are observed. Thus, depending on the complexity of the electronic configuration of the inert gas, the temperature of the gas in the plasma decreases and in the plasma of the Air + Xe mixture its value is in the range of 4500–6000 K.

It can be seen that the main compounds formed in this system are NO and NO^+^. The equilibrium concentration of NO in pure air and in mixtures of air with inert gases is at the same level and does not depend on the choice of inert gas. On the contrary, the equilibrium concentration of the NO^+^ ion in pure air is maximum and decreases in mixtures of air with inert gases. Moreover, the concentration of NO^+^ is the lower, the more complex the electronic configuration of the inert gas.

## 4. Conclusions

It was shown that the addition of He to air does not fundamentally change the spectral pattern of air. In contrast, the addition of Ar suppresses the N^+^ band at 463.0 nm, while the other bands of nitrogen ions slightly decrease. The addition of Kr leads to even greater suppression of the line intensities of nitrogen ions, as well as oxygen ions. It should be noted that when He, Ar, and Kr are added, the atomic oxygen line retains a low intensity. The addition of Xe results in complete suppression of the air component bands.

The formation of molecules in LIDB plasma can be summarized as follows.

1. The formation of NO and NO_2_ in LIDB plasma noticeably depends on the type of inert gas.
2. An increase in the concentration of NO and NO_2_ is affected by the complication of the electronic configuration of an inert gas, as well as a decrease in its ionization potential.
3. The complication of the electronic configuration of an inert gas, as well as a decrease in its ionization potential, leads to the suppression of the lines of nitrogen and oxygen ions. Thus, it can be assumed that the main mechanism affecting the chemical transformations in these mixtures and in particular, the formation of NO and NO_2_ is atomic.
4. The formation of ozone occurs outside the gas discharge zone by the photolytic reaction.
5. Using the method of thermodynamic analysis, it has been established that the equilibrium concentration of NO in in 1:1 mixtures of air with inert gases does not depend on the choice of an inert gas. On the contrary, the equilibrium concentration of the NO^+^ ion decreases as the electronic configuration of the inert gas becomes more complex.

In the future, it is planned to use this method on real systems, for example, for the treatment of abiogenic media obtained after irradiation with gas mixtures (for example, saline) with a further transition to blood treatment in order to increase its antioxidant potential.

## Figures and Tables

**Table 1:** Laser parameters.

Wavelength, µm	1.064
Radiation frequency, Hz	10
Energy per pulse, mJ	820
Pulse duration, ns	15
Area of a focal spot, cm^2^	10^−2^
Pulse power, MW	50
Irradiance, GW/cm^2^	5
Fluence, J/cm^2^	80
Photon flux density, cm^−2^ s^−1^	2.9 × 10^28^
Electric field strength, MV/cm	1.4

**Table 2:** Electronic configuration, ionization potentials, and characteristics of metastable states of Ar, He, Kr, and Xe [28].

Inert Gas	Electronic Configuration	Ionization Energy, eV	Metastable States	
			Excitation Energy, eV	Lifetime, s
Hе	1s^2^	24.6	19.82 (2^3^S_1_)	6 × 10^5^
Ar	1s^2^2s^2^2p^6^3s^2^3p^6^	15.8	11.55 (4^3^P^0^_2_) 11.72	>1.3 -
Kr	1s^2^2s^2^2p^6^3s^2^3p^6^ 3d^10^4s^2^4p^6^	14.0	9.91 10.5	- -
Xe	1s^2^2s^2^2p^6^3s^2^3p^6^ 3d^10^4s^2^4p^6^4d^10^5s^2^5p^6^	12.1	8.32 9.4	- -

**Table 3:** Comparative intensities of the lines of excited air particles.

Line (Band), nm	Transition	Air	Air/He = 9	Air/Ar = 1	Air/Kr = 1	Air/Xe = 1
N^+^ (399.5)	^1^D_2_-^1^P^o^_1_	strong	strong	strong	medium	weak
N^+^ (463.0)	^3^P_2_-^3^P^o^_2_	strong	strong	-	-	-
N^+^ (500.5)	^3^D_3_-^3^F^o^_4_	strong	strong	strong	medium	-
N^+^(568.0)	^3^D_3_-^3^P^o^_2_	medium	medium	medium	medium	-
N^+^ (594.1)	^3^D^o^_3_-^3^P_2_	medium	medium	medium	-	-
O (615.8)	^5^D^o^_4_-^5^P_3_	weak	weak	weak	weak	-
O^+^ (407.6)	^4^F_9/2_-^4^D^o^_7/2_	strong	strong	strong	-	-
O^2+^ (334.0)	^3^S_1_-^3^P^o^_2_	medium	medium	medium	-	-

## Data Availability

Data are available upon request.
